# Comparative analysis of monoaminergic cerebrospinal fluid‐contacting cells in *Osteichthyes* (bony vertebrates)

**DOI:** 10.1002/cne.24204

**Published:** 2017-03-29

**Authors:** Anna L. Xavier, Romain Fontaine, Solal Bloch, Pierre Affaticati, Arnim Jenett, Michaël Demarque, Philippe Vernier, Kei Yamamoto

**Affiliations:** ^1^ Paris‐Saclay Institute of Neuroscience (Neuro‐PSI), CNRS, Université Paris‐Sud, Université Paris‐Saclay Gif‐sur‐Yvette 91190 France; ^2^ TEFOR Core Facility, Paris‐Saclay Institute of Neuroscience (Neuro‐PSI), CNRS, Université Paris‐Sud, Université Paris‐Saclay Gif‐sur‐Yvette 91190 France; ^3^ Present address: Department of Glial Therapeutics Center for Basic and Translational Neuroscience, University of Copenhagen Copenhagen 2200 Denmark; ^4^ Present address: Department of Basic Sciences and Aquatic Medicine NMBU School of Veterinary Medicine Oslo 0033 Norway

**Keywords:** CSF‐contacting cells, dopamine, evolution, hypothalamus, monoamine, paraventricular organ, serotonin, ventricle, RRID: AB_2201528, RRID: AB_2314334, RRID: AB_477522, RRID: AB_94865, RRID: AB_221448, RRID: AB_2314655, RRID: AB_10000240, RRID: AB_87181

## Abstract

Cerebrospinal fluid‐contacting (CSF‐c) cells containing monoamines such as dopamine (DA) and serotonin (5‐HT) occur in the periventricular zones of the hypothalamic region of most vertebrates except for placental mammals. Here we compare the organization of the CSF‐c cells in chicken, *Xenopus*, and zebrafish, by analyzing the expression of synthetic enzymes of DA and 5‐HT, respectively, tyrosine hydroxylase (TH) and tryptophan hydroxylase (TPH), and draw an evolutionary scenario for this cell population. Due to the lack of TH immunoreactivity in this region, the hypothalamic CSF‐c cells have been thought to take up DA from the ventricle instead of synthesizing it. We demonstrate that a second TH gene (*TH2*) is expressed in the CSF‐c cells of all the three species, suggesting that these cells do indeed synthetize DA. Furthermore, we found that many CSF‐c cells coexpress *TH2* and *TPH1* and contain both DA and 5‐HT, a dual neurotransmitter phenotype hitherto undescribed in the brain of any vertebrate. The similarities of CSF‐c cells in chicken, *Xenopus*, and zebrafish suggest that these characteristics are inherited from the common ancestor of the *Osteichthyes*. A significant difference between tetrapods and teleosts is that teleosts possess an additional CSF‐c cell population around the posterior recess (PR) that has emerged in specific groups of *Actinopterygii*. Our comparative analysis reveals that the hypothalamus in mammals and teleosts has evolved in a divergent manner: placental mammals have lost the monoaminergic CSF‐c cells, while teleosts have increased their relative number.

## 1 Introduction

Cerebrospinal fluid‐contacting (CSF‐c) cells are a peculiar cell population with a bipolar morphology, one process protruding into the CSF. CSF‐c cells are present in various brain regions around the ventricles (Vígh et al., 2004). In nonmammalian species, CSF‐c cells located around the hypothalamic recess contain monoamines, such as dopamine (DA) and serotonin (5‐HT) (Ekström, Honkanen, & Steinbusch, 1990; Gonzalez & Smeets, 1994; Hirunagi, Hasegawa, Vigh, & Vigh‐Teichmann, 1992; Kaslin & Panula, 2001; Meek & Joosten, 1989; Meek, Joosten, & Steinbusch, 1989; Nakane et al., 2010; Reiner, Karle, Anderson, & Medina, 1994; Ueda, Nojyo, & Sano, 1984). These monoaminergic CSF‐c cells around the hypothalamic recess are not found in placental mammals (Smeets & Reiner, 1994), and their characteristics have not been well elucidated.

DA and 5‐HT are synthesized from the aromatic amino acids tyrosine and tryptophan, respectively, by rate‐limiting enzymes, tyrosine hydroxylase (TH), and tryptophan hydroxylase (TPH) (reviewed in Yamamoto & Vernier, 2011). TH and TPH belong to the same family of aromatic amino acid hydroxylase (AAAH). The corresponding metabolites, L‐3,4‐dihydroxyphenylalanine (L‐DOPA) and 5‐hydroxytryptophan (5‐HTP), are then transformed into DA and 5‐HT by the same enzyme, the amino acid decarboxylase (AADC). Thus, the difference between DA‐ and 5‐HT‐synthesizing cells solely depends on the presence of either TH or TPH, which determines the neurotransmitter phenotype.

There are two TPH‐encoding genes in vertebrates, *TPH1* and *TPH2* (Walther et al., 2003). *TPH2* is expressed in 5‐HT cell populations located in the raphe nuclei. In the mammalian brain, *TPH1* is expressed only in the pineal gland (epiphysis) that synthesises melatonin (MEL) (Walther & Bader, 2003). In contrast, nonmammalian species possess abundant *TPH1*‐expressing (*TPH1* ^+^) cell populations in the forebrain including the CSF‐c cells (Gaspar & Lillesaar, 2012; Kang, Thayananuphat, Bakken, & Halawani, 2007; Meneghelli et al., 2009).

Our previous study showed that two TH‐encoding genes, *TH1* and *TH2*, are present in *Osteichthyes*, except in placental mammals that have secondarily lost the *TH2* gene (Yamamoto, Ruuskanen, Wullimann, & Vernier, 2010). In the zebrafish CSF‐c cell populations, *th2* is much more abundantly expressed than *th1*, and the localization of the *th2* ^+^/*th1* ^–^ cells well matches that of so‐called DA‐accumulating cells (Yamamoto, Ruuskanen, Wullimann, & Vernier, 2011). Hypothalamic CSF‐c cells in nonmammals has been long considered to accumulate DA instead of synthesizing it (Smeets & Gonzalez, 1990; Smeets & Reiner, 1994), because they are DA‐immunopositive (DA^+^) but TH‐immunonegative (TH^–^). We found that the commercially available TH antibodies do not detect TH2 protein in immunohistochemistry (Yamamoto et al., 2010). These observations raise the possibility that the so‐called DA‐accumulating cells indeed synthetize DA using TH2 in nonmammalian vertebrates.

The current study addresses the evolution of the monoaminergic CSF‐c cells, as well as the evolution of the surrounding brain area, focusing on the two paralogous genes of DA and 5‐HT synthetic enzymes. Since the CSF‐c cells are located along the ventricular wall, their organization largely depends on the configuration of the hypothalamic recess. We thus took into account the ventricular morphology in our analysis, as we recently did to study the regionalization of the optic recess region (ORR) (Affaticati et al., 2015).

We demonstrate that, as it is the case in zebrafish, *TH2* is expressed in the CSF‐c cells known as the “DA‐accumulating cells” in chicken and *Xenopus*. Moreover, we found that many of these *TH2* cells coexpress *TPH1*, also in all three species. Our study suggests that this dual phenotype in the monoaminergic CSF‐c cells is inherited from the common ancestor of *Osteichthyes*. Nonetheless, a large difference is also observed between tetrapods and teleosts. The CSF‐c cells in tetrapods are organized around one hypothalamic recess, while those in teleosts are organized around two hypothalamic recesses.

## 2 Materials and methods

### 2.1 Animals and tissue preparation

In this study we used three vertebrate species: chicken (*Gallus gallus*), *Xenopus* (*Xenopus tropicalis*), and zebrafish (*Danio rerio*). All experimental protocols and handling, use, and care of laboratory animals were conducted in compliance with the current normative standards and approval of the French Government (reference document n°APAFIS#1286‐ 2015062616102603 v5, AP 2014‐28 V2 mars).

Embryonic (E15; *n* = 3) and posthatched chicken (day 0; *n* = 2 and 4 weeks; *n* = 5; sex unidentified) were purchased from EARL Morizeau (Dangers, France). Animals were deeply anesthetized with a mixture of ketamine (60 mg/kg) and xylazine (10 mg/kg). They were transcardially perfused with phosphate buffer saline (PBS, Fisher Scientific, Fair Lawn, NJ), followed by ice‐cold 4% paraformaldehyde (PFA, Electron Microscopy Sciences, Hatfield, PA) in PBS. Brains were dissected and postfixed overnight at 4°C in 4% PFA in PBS containing 0.1% of Tween 20 (Fisher Scientific; PBST).

*Xenopus* brains were obtained from adult individuals (1–3 years; *n* = 5; males), kindly provided by Dr. Muriel Perron (Neuro‐PSI, Université Paris‐Saclay). Animals were deeply anesthetized in tricaine methanesulfonate (MS222; Sigma‐Aldrich Co. LLC, St. Louis, MO) diluted in water (0.1%), decapitated, and the skull was removed to expose the brain. Brains were then fixed overnight at 4°C by immersion in ice‐cold 4% PFA in PBST.

Zebrafish were kept in our own colony. Adult brains (3 months to 2 years old animals; *n* = 30; both males and females) were used in all the experiments except for the whole brain imaging, in which 6‐week‐old zebrafish brains were used. Animals were either wild‐type (AB) or *ETvmat2*:*GFP*, an enhancer trap transgenic line in which the reporter gene is inserted into the second intron of vesicular monoamine transporter 2 (*vmat2*) (Kawakami et al., 2004; Wen et al., 2008). Zebrafish were deeply anesthetized using MS222 diluted in fish water (0.2%). Brains were then dissected and fixed in ice‐cold 4% PFA in PBST overnight at 4°C. For DA and 5‐HT double labeling, brains were fixed with 4% PFA in Tris‐buffered saline (TBS; 50 mM Tris‐HCl/150 mM NaCl) containing 1% sodium metabisulfite (SMB) and 0.1% of Tween 20 (TBS/SMB‐T; pH 7.2–7.5) overnight at 4°C (Yamamoto et al., 2011).

Brains from all species were dehydrated in ethanol (VWR Chemicals, VWR International) gradient series, and kept at −20°C in methanol (VWR Chemicals) until tissue processing. The brains were rehydrated prior to sectioning with vibratome (Leica VT 1000 S, Leica Biosystems GmbH, Nussloch, Germany), and frontal or sagittal sections (50–100 µm) were obtained for immunohistochemistry or in situ hybridization. When the experiment was not performed immediately after sectioning, the tissues were further dehydrated in ethanol gradient series and kept at −20°C in 100% ethanol or methanol until used.

### 2.2 Clarity

Samples were subjected to tissue clearing following CLARITY/PACT protocol, with some tissue‐specific adaptations (Chung et al., 2013; Mayrhofer et al., 2017; Treweek et al., 2015): dissected brains were fixed in freshly prepared ice‐cold methanol‐free PFA 4% in PBS (pH 7.4) overnight at 4°C. Samples were then infused in a precooled solution of freshly prepared hydrogel monomers (0.01 M PBS, 0.25% VA‐044 initiator, 5% dimethyl sulfoxide, 1% PFA, 4% acrylamide, and 0.0025% bis‐acrylamide) for 2 days at 4°C. After degassing the samples, the hydrogel polymerization was triggered by replacing atmospheric oxygen with nitrogen in a desiccation chamber for 3 hr at 37°C. Passive tissue clearing was performed at 37°C for 8 days in the clearing solution (8% SDS, 0.2 M boric acid, pH adjusted to 8.5) under gentle shaking. Subsequently the samples were thoroughly washed in PBST at room temperature with gentle shaking for 2 days.

### 2.3 Immunohistochemistry

Histological sections were rehydrated in ethanol gradient series and extensively washed in PBST at room temperature. Sections were incubated for 1 hr at room temperature with 4% normal goat serum (Bethyl Laboratories Inc., Montgomery, TX) in PBST containing 0.3–0.6% Triton X‐100 (Sigma‐Aldrich), and then incubated with primary antibodies (Table 1) overnight at 4°C. For double DA and 5‐HT immunolabeling, brains were treated in TBS/SMB‐T instead of PBST, for all the procedures until the primary antibody incubation.

**Table 1:** List of primary antibodies

**Antigen**	**Immunogen**	**Manufacturer**	**Species**	**Dilution**	**Reference (RRID#)**
Tyrosine hydroxylase (TH)	Tyrosine hydroxylase purified from PC12 cells	Millipore (Darmstadt, Germany; Catalog # MAB318)	Mouse (monoclonal)	1:500	Yamamoto et al. (2010) (AB_2201528)
Dopamine (DA)	Dopamine‐glutaraldehyde‐BSA conjugates	H.W.M. Steinbusch (Maastricht, The Netherlands)	Rabbit (polyclonal)	1:500	Yamamoto et al. (2011) (AB_2314334)
Serotonin (5‐HT)	Serotonin‐creatinine sulfate complex‐ BSA conjugates	Sigma Aldrich (St. Louis, MO, USA; Catalog # S5545)	Rabbit (polyclonal)	1:500	Bosco et al. (2013) (AB_477522)
Serotonin (5‐HT)	serotonin conjugated to BSA	Millipore (Catalog # MAB352)	Rat (monoclonal)	1:100	Bosco et al. (2013) (AB_94865)
HuC/D (16A11)	Human HuC/HuD neuronal protein	Invitrogen (Catalog # A‐21271)	Mouse (monoclonal)	1:500	Affaticati et al. (2015) (AB_221448)
HuC/D		B. Zalc (Salpêtrière Hospital, Paris, France)	Human (polyclonal)	1:2000	Alunni et al. (2013) (AB_2314655)
Green fluorescent protein (GFP)	Purified recombinant green fluorescent protein (GFP) emulsified in Freund's adjuvant	Aves Labs, Inc. (Tigard, OR, USA; Catalog # GFP‐1020)	Chicken (polyclonal)	1:500	Alunni et al. (2013) (AB_10000240)
ZO‐1	Human recombinant ZO‐1 fusion protein encompassing amino acids 334‐634	Invitrogen (cat # 339100)	Mouse	1:200	Zhang et al. (2010) (AB_87181)

Immunolabeling was revealed by an incubation with appropriate secondary antibodies conjugated to fluorophores (Alexa Fluor 1:1000; Molecular Probes‐Invitrogen/Thermo Fisher Scientific Inc.), either for 2 hr at room temperature or overnight at 4°C. DAPI (4′,6‐diamidino‐2‐phenylindole dihydrochloride, 1:1000; Sigma‐Aldrich) was used for nuclear counterstaining, and brain sections were mounted on slides using Fluoromount‐G Mounting Medium (Southern Biotech, Birmingham, AL).

CLARITY‐processed brains were incubated in blocking solution (PBST, 1% TritonX100, 10% dimethyl sulfoxide, 10% normal goat serum, 0.05 M glycine) overnight at 4°C. Subsequently samples were incubated in CLARITY‐staining solution (PBST, 0.1% Triton X100, 20% dimethyl sulfoxide, 2% normal goat serum, 0.05% azide) with primary antibody (either chicken anti‐GFP at 1:400, or mouse anti‐TH at 1:600) for 7 days at room temperature under gentle shaking. After four washing steps in PBST, samples were incubated in staining solution with secondary antibody (either goat anti‐chicken Alexa Fluor 488 at 1:400 or goat anti‐mouse Alexa Fluor 488 at 1:400) for 7 days at room temperature. Samples were washed for 2 days in PBST. For 3D segmentation of the brain ventricle in *ETvmat2*:*GFP* fish line, the brains were labeled with DiI (DiIC_18_(3) Stain, Molecular Probes). 1 mM Dil stock solution was prepared by dissolving DiI powder in DMSO. Brains were incubated for 2 days in the CLARITY‐staining solution containing 1 µM DiI.

### 2.4 In situ hybridization

#### 2.4.1 Probe synthesis

Following the general formatting of gene symbols, chicken genes will be abbreviated with upper‐case letters *(*e.g., *TH1)*, while those in *Xenopus* and zebrafish will be abbreviated with lower‐case letters (e.g., *th1)*. In order to refer to the homologous genes throughout the three species or throughout vertebrates, we will use upper‐case (e.g., *TH1*) in this article.

Chicken and *Xenopus TH* and *TPH* genes were cloned into pCRII Vector (Invitrogen/Thermo Fisher Scientific Inc.) or StrataClone (Agilent Technologies, Santa Clara, CA), after PCR amplification of the transcripts using specific primers (Table 2). Zebrafish *th2* and *tph1a* had already been used in previous publications (Bellipanni, Rink, & Bally‐Cuif, 2002; Yamamoto et al., 2010, 2011). Antisense and sense RNA probes were synthesized by in vitro transcription using T3, T7, or Sp6 RNA polymerase (Promega, Madison, WI) and labeled with fluorescein‐12‐UTP or digoxigenin‐11‐UTP (Sigma‐Aldrich Co. LLC./Roche). Probes were purified using Nucleospin RNA clean‐UP kit (Macherey‐Nagel, Hoerdt, France) and analyzed by gel electrophoresis to confirm the size.

**Table 2:** List of probes synthesized for in situ hybridization

**Gene**	**Species**	**Target sequence**	**Primers**
Tyrosine hydroxylase 1 (*TH1*)	*Gallus gallus*	NM 204805.2	Forward CATGTCTCCACGGTTCATTGReverse GACATGCCCCAGTAGCTCAT
Tyrosine hydroxylase 2 (*TH2*)	*Gallus gallus*	XM 001235000.3	Forward CTCTGCCGATGATTTTGATGReverse TCTATGCTGCTGGTGAATGG
Tryptophan hydroxylase 1 (*TPH1*)	*Gallus gallus*	NM 204956.1	Forward CAAGCTTTACCCAACTCATGCReverse TGGACCTCTCACTCTTCCATT
Tyrosine hydroxylase 1 (*th1*)	*Xenopus tropicalis*	ENSXETG00000014030	Forward CGGACCTGGATTTAGAGCACReverse CTCTTAGCCCGTCCAGAGAA
Tyrosine hydroxylase 2 (*th2*)	*Xenopus tropicalis*	ENSXETG00000025926	Forward AGGATCACCCTGGATTTGGTReverse GTTCCTCAGCTCGGCTTTTA
Tryptophan hydroxylase 1 (*tph1*)	*Xenopus tropicalis*	ENSXETG00000018984	Forward AACGACAATGTCTGCGAGAGReverse CAAGCCAATTTCTTGGGAGA

#### 2.4.2 In situ hybridization

Fluorescent in situ hybridization was performed as described previously (see Fontaine et al., 2013) with modifications. Briefly, brain sections were rehydrated through a series of ethanol solutions, and permeabilized by a 10‐min treatment with proteinase K (10 µg/ml; Sigma‐Aldrich Co. LLC.). Samples were first incubated in hybridization buffer for 4 hr at 65°C, then hybridization was performed by incubating with a mixture of probes (2 ng/µl for each probe) for 18 hr at 65°C. Samples were then washed (gradient posthybridization washes with 50% formamide (Fisher Scientific)/50% 2X saline sodium citrate buffer (SSC; Sigma‐Aldrich Co. LLC.), 2X SSC, 0.2X SSC, and PBST), treated for 30 min with 3% H_2_O_2_ (Sigma‐Aldrich Co. LLC.) to inactivate endogenous peroxidases, and rinsed in PBST. Probes were detected as follows: (1) incubation with hapten‐specific antibodies conjugated to digoxigenin (DIG) and/or to fluorescein (Sigma‐Aldrich Co. LLC./Roche); (2) incubation in 0.001% H_2_O_2_ with suitable fluorophore‐conjugated tyramide (TAMRA and/or FITC) and (3) peroxidase inactivation, by 2% H_2_O_2_. After extensive washes, samples were counterstained with DAPI.

For nonfluorescent in situ hybridization, brain tissue sections were incubated with anti‐DIG‐alkaline phosphatase (DIG‐AP; Roche Diagnostics) after posthybridization washes, and the signal was visualized by incubation with nitroblue tetrazolium chloride (NBT; 4.5 µl/ml) and 5‐bromo‐4‐chloro‐3‐indolylphosphate (BCIP; 3.5 µl/ml) in a TNM buffer (Tris‐HCl, pH9.5, 0.1 M NaCl, 0.05 M MgCl_2_ in H_2_O).

In some cases, in situ hybridization was coupled to immunohistochemistry, as described above.

### 2.5 Image acquisition

Brain sections were imaged using a Zeiss LSM700 laser scanning confocal microscope with 20X (numerical aperture (NA) 1.30) and 40X oil immersion (NA 1.25) objective lens (Zeiss). For all confocal images, stacks were acquired using 1 µm‐Z steps. Acquired images were adjusted for brightness and contrast using FIJI/ImageJ software.

CLARITY‐treated brains were mounted in a fructose‐based high refractive index solution (fHRI) prepared as follows: 70% fructose, 20% DMSO in 0.002 M PBS, 0.005% sodium azide. The refractive index of the solution was adjusted to 1.4571 using a refractometer (Krüss GmbH, Hamburg, Germany). In preparation for imaging, samples were incubated in 50% fHRI/50% PBST for 6 hr and finally incubated in 100% fHRI for at least 12 hr. For imaging, samples were mounted in 1% low melting point agarose and covered with fHRI. Whole‐mount brain fluorescence was captured using a Leica TCS SP8 laser scanning confocal microscope equipped with a Leica HC FLUOTAR L 25x/1.00 IMM motCorr objective. Fluorescence signal was detected by exciting the fluorophores with lasers at wavelength of 488 and 552 nm. Detection was performed by two internal photomultipliers (PMT).

### 2.6 3D whole brain reconstruction

The 3D‐visualizations of zebrafish specimens were generated using amira6 3D visualization and reconstruction software platform (FEI, Hillsboro, OR) on a HP Z720 workstation.

In the process of direct volume rendering, the brightness value of each voxel (volume pixel) is converted into an opacity value. Bright voxels therefore are rendered very opaque, while dark voxels are rendered very transparent. This allows 3D rendering of dense data by promoting the visibility of bright structures over the dark background. To facilitate the differentiation between different levels of expression, we applied multicolored colormaps to the data: in the *vmat2*‐GFP channel, the lowest intensity values are visualized in blue and the highest in green (volrenGreen), while for the channel reflecting the TH immunoreactivity, lowest intensity values are in orange and the highest intensity values are in yellow (volrenGlow).

To visualize the relationship of the *vmat2*‐GFP expression pattern (green) with the ventricle system (magenta), we applied manual‐ and threshold‐based segmentation and surface reconstruction. While we were able to define the volume of the *vmat2*‐GFP expression pattern using amira's build‐in thresholding and surface‐simplification algorithms, the ventricle system was segmented manually on the basis of our reference staining (DiI). Since DiI in our application stains all the membranes, we were able to reconstruct the morphology of the ventricles by following the high‐density staining (presumably tight junctions) of the cells facing the lumen of the ventricle as well as the void of the ventricles itself. Subsequently the manual segmentation was refined by thresholding and supervised intensity‐based region growing. Surfaces were computed (SurfaceGen) on the basis of the defined volumes. The segmentation and resulting surfaces were smoothed and simplified with the appropriate tools in amira (Segmentation Editor/Smooth label; Simplification Editor).

## 3 Results

### 3.1 Distribution of the CSF‐c cells in relation to the ventricular morphology

**Figure 1:**
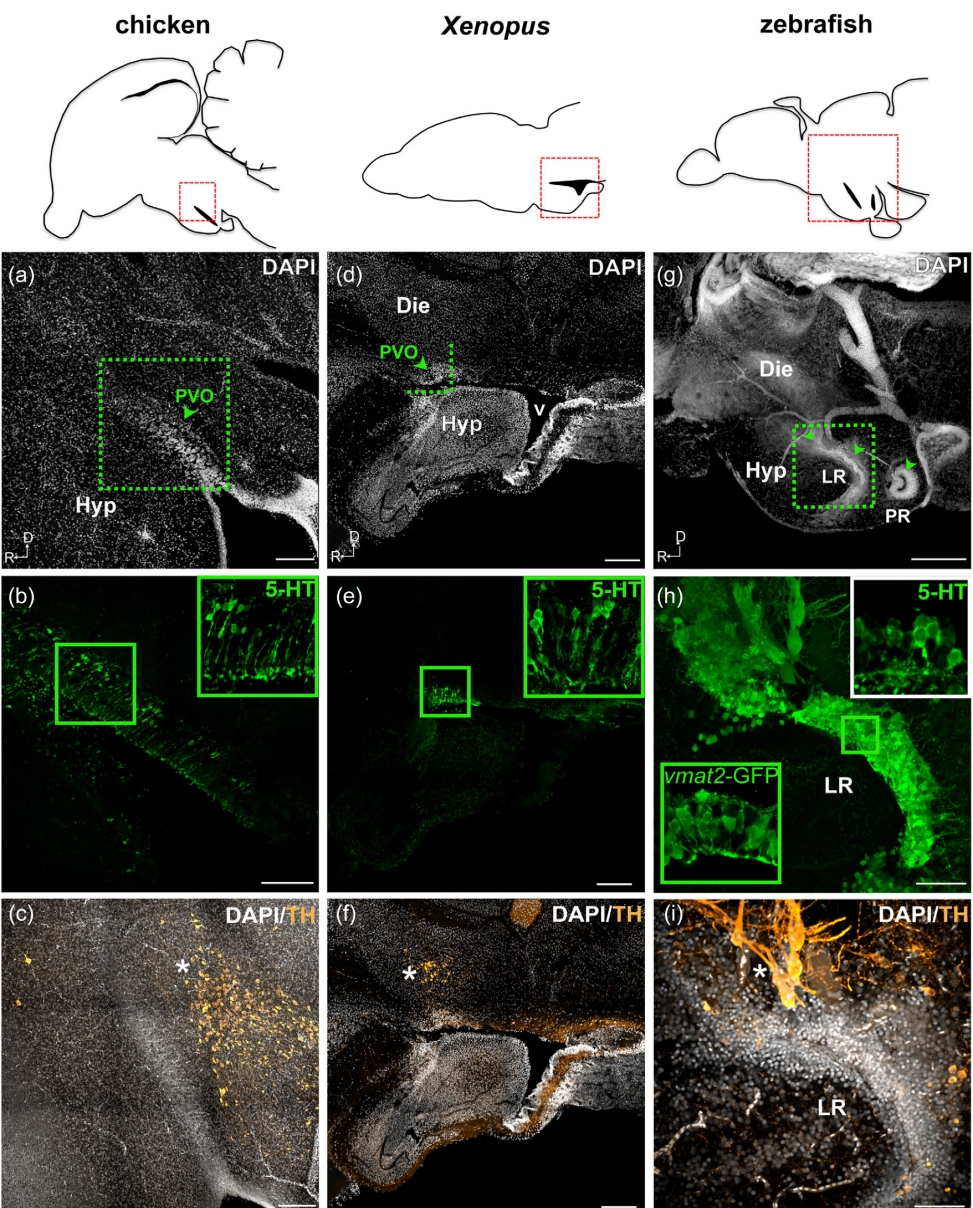
Monoaminergic CSF‐c cells of chicken, *Xenopus*, and zebrafish in relation to the organization of the hypothalamic ventricle. Schematic drawings of sagittal sections of chicken, *Xenopus*, and zebrafish brains are shown in the top panels, and for each species, micrographs of the red squared areas are shown below (a–c for chicken, d–f for *Xenopus*, and g–i for zebrafish). DAPI staining (gray) delineates the recesses of the hypothalamic ventricle. Confocal images (Z‐projection = 10 µm) with higher magnification obtained from the area depicted in (a) (dashed green square) show 5‐HT^+^ CSF‐c cells (green) aligned along the dorsal side of the hypothalamic recess (b; inset at higher magnification). TH immunoreactive cells (orange) are not observed within the PVO (arrowhead), but are abundant in the area dorsocaudal to it (c; asterisk). In the *Xenopus* sagittal section close to the midline, PVO (d; arrowhead) is observed at the anterior edge of the large ventricle (v). The PVO is visualized with 5‐HT^+^ CSF‐c cells (e; green; inset at higher magnification). TH immunoreactive cells (orange) are observed dorsal to the PVO (f; asterisk). In zebrafish, three CSF‐c cell populations (locations indicated by arrowheads in g) are located around two hypothalamic recesses. The two anterior CSF‐c cell populations are located in front of and around the lateral recess (LR), while the posterior population surrounds the posterior recess (PR). Higher magnification of the squared area in (g) is shown in (h) and (i) (Z‐projection = 10 µm). CSF‐c cells revealed by the expression of GFP in the enhancer trap transgenic line *ETvmat2*:*GFP* (*vmat2*‐GFP*;* green inset) are lined along the ventricular zone (h). The white inset in (h) shows the 5‐HT labeling in the same area (the image is taken from a different sample). TH immunoreactive cells (orange) are found dorsal to the LR (i; asterisk). D = dorsal; Die = diencephalon; Hyp = hypothalamus; LR = lateral recess; PR = posterior recess; PVO = paraventricular organ; R = rostral; v = ventricle. Scale bar = 200 µm in (a–g); 50 µm in (h, i)

Monoaminergic CSF‐c cells are located along the ventricle in the hypothalamic area. Their organization therefore depends on the shape of the hypothalamic recess. Tetrapods such as chicken (Figure 1a–c) and *Xenopus* (Figure 1d–f) have one hypothalamic recess, along which the CSF‐c cells are located. The cluster of CSF‐c cells are called the paraventricular organ (PVO; Figure 1a,b,d,e), and they are lined up along the ventricular wall, with their processes touching the ventricular surface (CSF‐c cells are visualized with 5‐HT immunolabeling (5‐HT^+^) in Figure 1).

**Figure 2:**
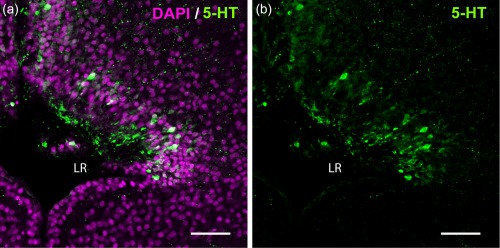
5‐HT^+^ CSF‐c cells in the *Xenopus* PVO. The laterally extended hypothalamic recess (lateral recess; LR) is visualized with DAPI staining (magenta) from a frontal section (midline to the left). CSF‐c cells immunolabeled for 5‐HT (green) are located medially in the rostral hypothalamus and laterally in the caudal hypothalamus. (a) depicts both DAPI and 5‐HT stainings, while (b) shows 5‐HT only (same picture). In the projection of confocal images (15 µm), the rostromedial and caudolateral CSF‐c cells look continuous. Scale bar = 50 µm

In amniotes, the hypothalamic recess is thin and morphologically indistinguishable from the diencephalic part of the third ventricle. In amphibians the hypothalamic recess is much larger (Figure 1d), and it is called the “lateral recess of the infundibulum” (Neary & Northcutt, 1983) due to its lateral extension (Figure 2a). CSF‐c cells are located in the rostromedial (shown in the section close to the midline; Figure 1d,e) and caudolateral parts of the recess. Based on the projections of confocal image stacks from frontal sections, the rostromedial and caudolateral 5‐HT^+^ CSF‐c cells appear to be continuous (Figure 2b).

**Figure 3:**
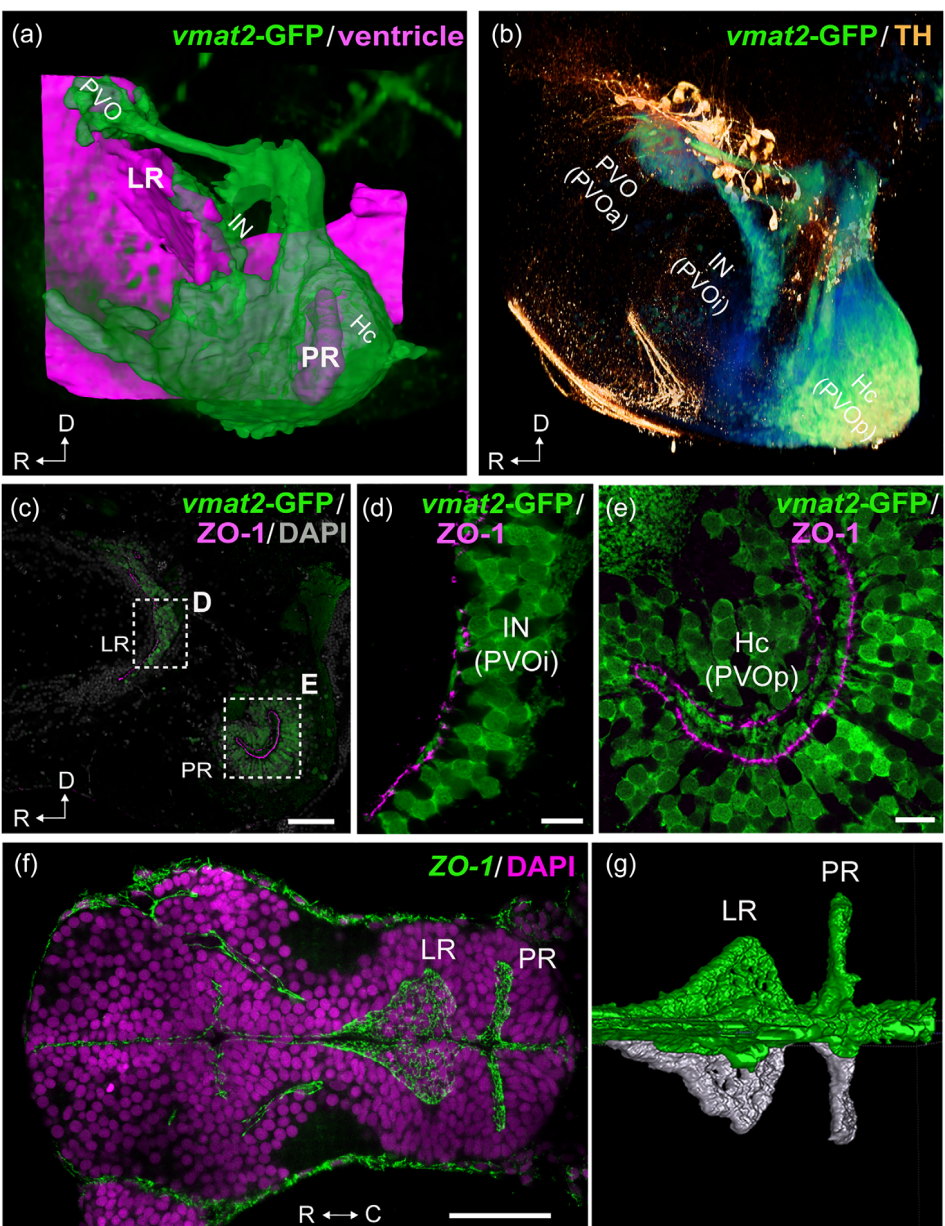
Organization of the two hypothalamic recesses in zebrafish. The hypothalamic area of adult (a–e; sagittal view) and embryonic (f, g; horizontal view) zebrafish brains are shown. The anterior is to the left in all figures. 3D visualization of the images of (a) and (b) are shown in the Supporting Information Figures S1 and S2 respectively. (a) The three *vmat2*‐GFP CSF‐c cells (green; PVO, IN, and Hc) are organized around the two hypothalamic recesses, namely the lateral recess (LR) and the posterior recess (PR). The CSF‐c cells (green) are visualized using an enhancer trap *vmat2*:*GFP* zebrafish line, and the ventricular zone (magenta) is reconstructed from ventricular surfaces delineated by DiI staining. (b) Immunolabeling for *vmat2*‐GFP (green) and TH (orange). Prominent TH‐immunopositive cells (orange) are found dorsal to the CSF‐c cells populations (green): PVO, IN, and Hc, which are alternatively named PVOa, PVOi, and PVOp. (c–e) Adult hypothalamic recesses double labeled for *vmat2*‐GFP and ZO‐1. The areas delimited by dashed rectangles in (c) are shown in (d**)** and (e), which demonstrate the end feet of the process of CSF‐c cells bathing in the ventricle. (f, g**)** The ZO‐1 immunolabeling in the developing brain (48 hr postfertilization), demonstrating that the LR and PR are separate ventricular extension from early embryonic stages. (g) shows 3D semiautomatic segmentation of the ZO‐1 immunolabeling in the LR and PR shown in (f). The right hemisphere and the midline are visualized in green, while the left hemisphere is visualized in white, to highlight the intricate ventricular organization. C = caudal; D = dorsal; Hc = caudal zone of periventricular hypothalamus; IN = intermediate nucleus of hypothalamus; LR = lateral recess; PR = posterior recess; PVO = paraventricular organ; PVOa = anterior paraventricular organ; PVOi = intermediate paraventricular organ; PVOp = posterior paraventricular organ; R = rostral. Scale bars = 50 µm in (c**)** and (f); 10 µm in (d) and (e)

In zebrafish, three CSF‐c cell populations are organized along two hypothalamic recesses, instead of the one observed in tetrapods: the lateral recess (LR) and the posterior recess (PR) (Figures 1g,h and 3, Supporting Information Figures S1 and S2). In an enhancer trap *vmat2*:*GFP* line which labels cells that express the vesicular monoamine transporter 2 (*vmat2*), GFP‐immunoreactivity was observed in three CSF‐c cell populations (*vmat2*‐GFP; Figures 1h and 3a–e). The anterior CSF‐c cell population is located in front of the LR, the intermediate one is around the LR (Figures 1g,h and 3a–d, Supporting Information Figure S1), and the posterior population completely surrounds the PR (Figure 3a,c,e, Supporting Information Figure S1). The PR is a clearly distinct ventricular system separated from the LR. ZO‐1 immunostaining (which labels tight junctions of neuroepithelial cells lining the ventricle) in the embryonic brain shows that the LR and PR are already formed at 48 hr postfertilization (Figure 3f,g; Affaticati et al., 2015).

In all the three species examined, sagittal sections close to the midline show prominent TH^+^ cells that are located dorsal to the CSF‐c cells (Figures 1c,f,i and 3b, Supporting Information Figure S2).

### 3.2 *TH2* is expressed in the hypothalamic CSF‐c cells in chicken and *Xenopus*

Previous results have shown that *TH2* is found in the genome of various nonmammalian vertebrate species (Yamamoto et al., 2010). Although well established in teleosts (Chen, Priyadarshini, & Panula, 2009; Filippi, Mahler, Schweitzer, & Driever, 2010; Yamamoto et al., 2010, 2011), the expression of the *TH2* gene has not been demonstrated in the tetrapod CSF‐c cells. We hypothesized that the so‐called “DA‐accumulating cells” described in nonmammals, based on the presence of DA immunoreactivity (DA^+^) but the absence of TH immunoreactivity (TH^–^) (Smeets & Gonzalez, 1990; Smeets & Reiner, 1994), are in fact “DA‐producing cells,” synthetizing this monoamine using the tyrosine hydroxylase encoded by *TH2*.

**Figure 4:**
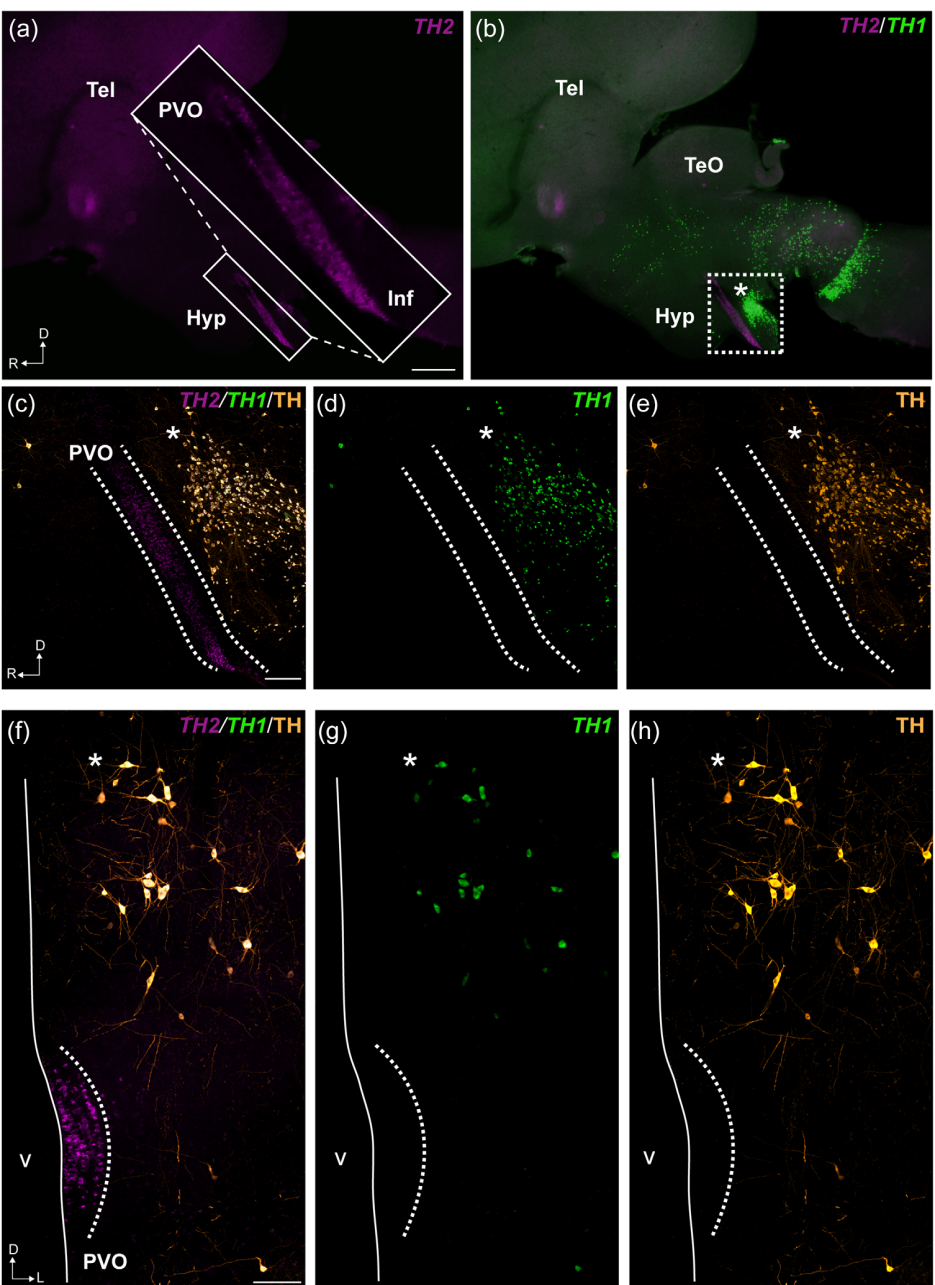
Localization of *TH1*, *TH2*, and TH immunoreactivity in the PVO of chicken. Representative micrographs of chicken brains in sagittal sections (a–e) and frontal sections (f–h) are shown. Abundant labeling of *TH2* transcripts is found all along the hypothalamic recess (a; magenta). In the inset of (a), the expression of *TH2* by PVO CSF‐c cells is shown at higher magnification. Double labeling with *TH1* (green) shows that there is no overlap for the expression of the two genes in the PVO (b). Confocal images (Z projections = 10 µm) of the squared area in (b) are shown in (c–e). *TH2* transcripts are localized along the PVO, in the area delimited by dashed lines (c). In contrast, *TH1* is absent in the PVO (d), as are TH immunoreactive cells (e; orange). The micrograph of the frontal section (ventricular zone to the left) also shows that the PVO contains *TH2* transcripts (f; magenta), but not *TH1* (g; green) nor TH immunoreactive cells (h; orange). *TH1* expressing cells and TH immunoreactive cells are observed dorsally to the PVO (asterisks in b–h). D = dorsal; Hyp = hypothalamus; Inf = infundibulum; L = lateral; R = rostral; Tel = telencephalon; TeO = optic tectum; v = ventricle. Scale bars = 1 mm in (a) (applies to a, b); 200 µm in (c) (applies to c, d, e); 100 µm in (f) (applies to f, g, h)

In agreement with this hypothesis, we show that *TH2* transcripts are found in CSF‐c cells in the chicken hypothalamus (Figure 4a,c,f). In chicken, *TH2* is expressed in the CSF‐c cells all along the hypothalamic recess, rostrally in the area identified as the PVO and extending caudally toward the infundibulum (Inf) (Figure 4a,c). The CSF‐c cells are devoid of *TH1* expression (Figure 4b,d,g) and of TH immunoreactivity (Figure 4e,h).

**Figure 5:**
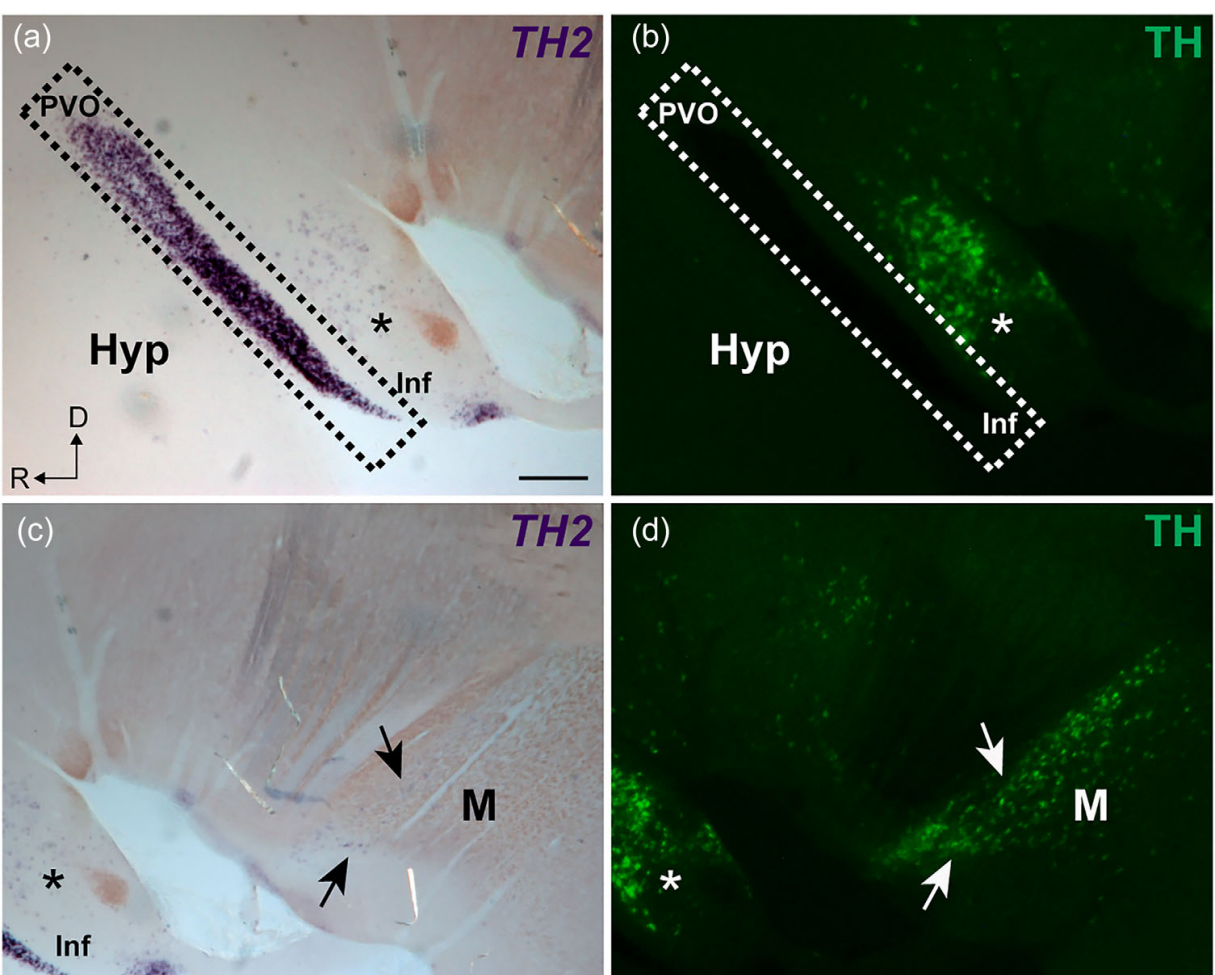
Double labeling for *TH2* nonfluorescent in situ hybridization and TH immunofluorescence in chicken. A sagittal section of chicken brain showing TH2 transcripts (purple) and TH immunoreactivity (green) in the hypothalamic (a, b) and mesencephalic (c, d) areas. *TH2* transcripts are abundant in the PVO (a; dashed area) and scarce in a cell population corresponding to A11 (a; asterisk). In contrast, TH immunoreactive cells are abundant in the A11 cell group (b; asterisk), while they are absent in the PVO (b; dashed area). The mesencephalic TH immunoreactive cells also express *TH2* (c; arrows), but compared to the TH immunoreactivity (d; arrows), the *TH2* ^+^ signal is very low. D = dorsal; Hyp = hypothalamus; Inf = infundibulum; M = mesencephalon; R = rostral. Scale bar = 200 µm in (a) (applies to a, b, c, d)

We observed low levels of *TH2* transcripts in cells located dorsolaterally to the CSF‐c cells (Figure 5a; asterisk). This cell population is described as the A11 dopaminergic cells in pigeon (Reiner et al., 1994; Figures 1c,4b–h,5b; asterisks). Besides, weak *TH2* labeling is observed in some cells of the mesencephalic DAergic population (Figure 5c; arrows), most of which also exhibit high levels of *TH1* ^+^ and TH^+^ (Figure 5d; arrows).

**Figure 6:**
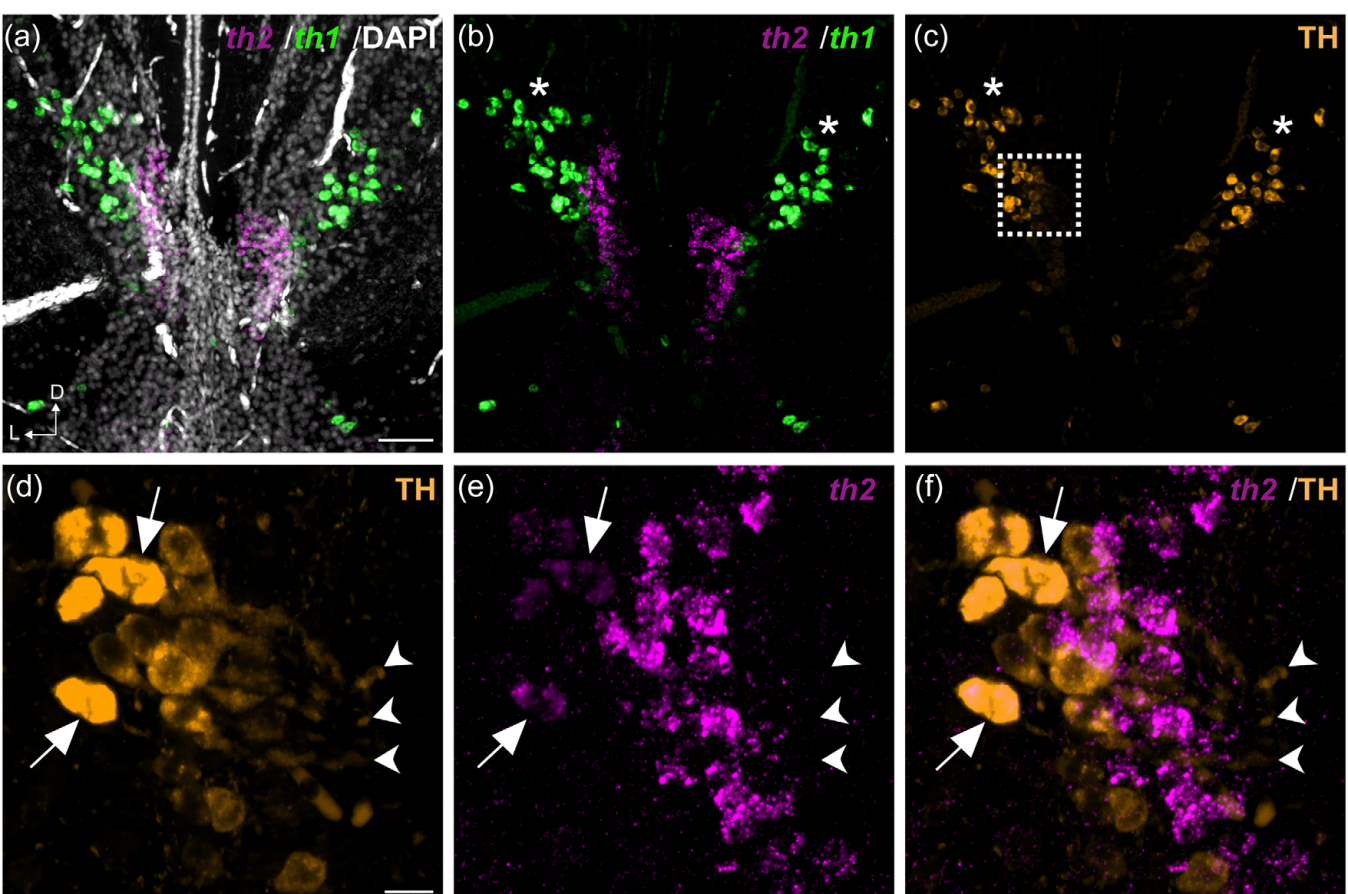
Localization of *th1*, *th2*, and TH immunoreactivity in the PVO of *Xenopus*. Confocal images obtained from a frontal section of the *Xenopus* PVO are shown (Z projections = 10 µm). Counterstaining with DAPI (gray) shows that *th2* (magenta) is expressed in the region corresponding to PVO (a). A cell population displaying strong *th1* signal (green) is found dorsolaterally to the PVO (asterisks in b), which overlaps with TH immunoreactivity (orange; asterisks in c). Higher magnification of the area delimited by a dashed square in (c) is shown in (d–f). Faint TH immunoreactivity is also found in CSF‐c cells in the *Xenopus* PVO. TH^+^ labeling is observed in the soma and in the processes bathing the ventricle of CSF‐contacting cells (arrowheads in d–f). A few CSF‐contacting cells expressing *th2* are also TH immunoreactive (arrows in d–f). D = dorsal; L = lateral. Scale bar = 50 µm in (a) (applies to a, b, c); 100 µm in (d**)** (applies to d, e, f)

**Figure 7:**
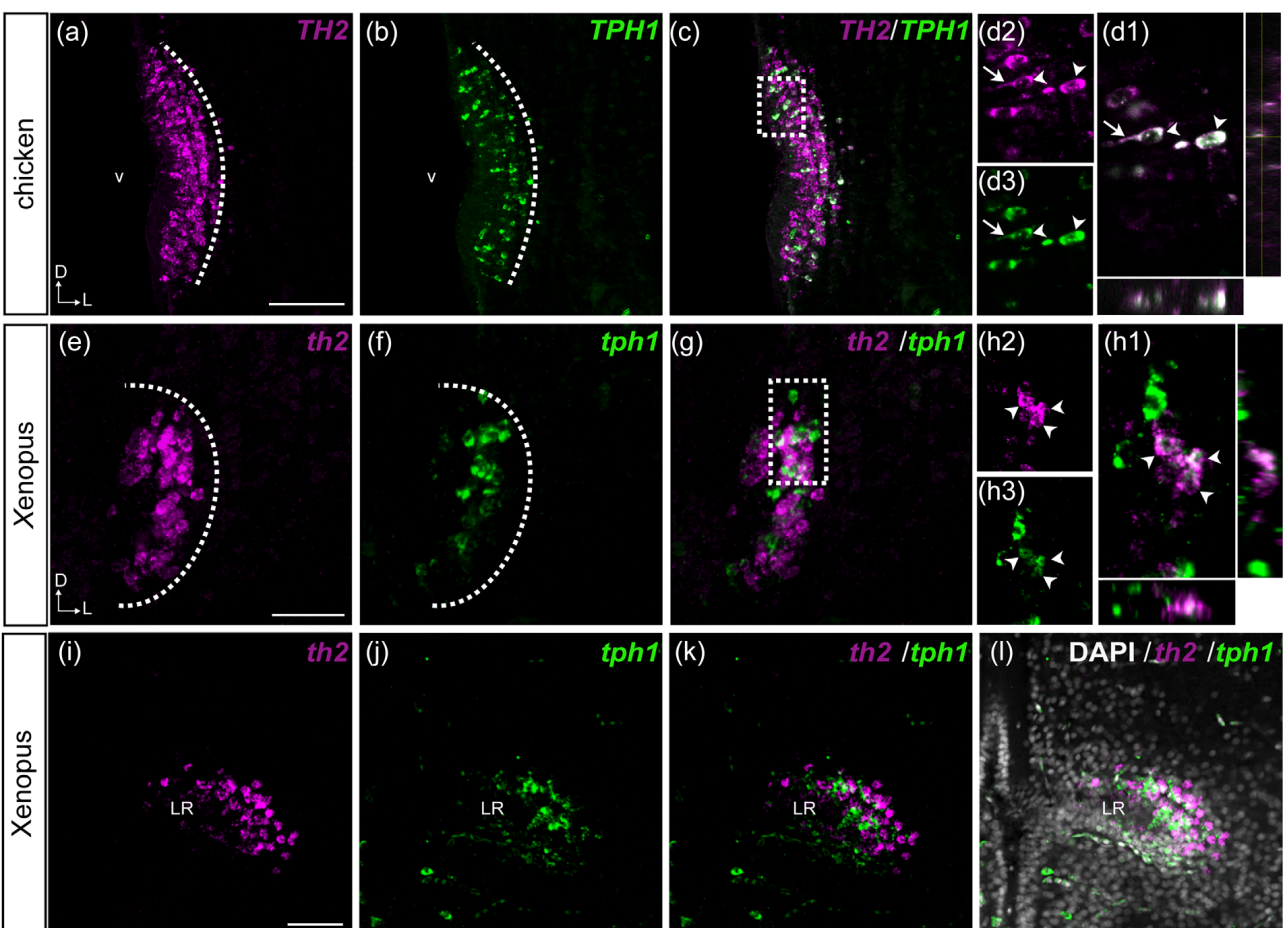
Colocalization of *TH2* and *TPH1* in the PVO of chicken and *Xenopus*. Frontal sections obtained from the PVO of chicken (a–d) and *Xenopus* (e–l) are shown. *TH2* (a; magenta) and *TPH1* (b; green) transcripts are observed in the same region of the chicken PVO, and some CSF‐c cells coexpress both genes (c, d; white). The area depicted by a dashed rectangle in (c) is shown in (d1–d3**)** at higher magnification. Optical sectioning by confocal microscopy shows that *TH2* (d2) and *TPH1* (d3) are expressed in the soma (arrowheads) and in the processes of CSF‐contacting cells (arrow). Orthogonal views of optical sections confirm the overlap of *TH2* and *TPH1* signals. In *Xenopus*, *th2* (e, i; magenta) and *tph1* (f, j; green) are expressed both in the rostromedial (e–h) and the caudolateral (i–l) parts of the PVO. Some CSF‐c cells coexpress both genes (g, k). The coexpression is confirmed by orthogonal views of optical sections (h1) of the area depicted by a dashed rectangle in (g), showing that *th2* (h2) and *tph1* (h3) are colocalized in the same cells (arrowheads). D = dorsal; L = lateral; LR = lateral recess; v = ventricle. Scale bar = 100 µm in (a) (applies to a, b, c) and in (e) (applies to e, f, g); 50 µm in (i**)** (applies to i, j, k, l)

The *th2* transcripts are located in the CSF‐c cells in the ventricular zone of the *Xenopus* forebrain, overlapping with the area identified as the PVO in amphibian species (Gonzalez & Smeets, 1994; Neary & Northcutt, 1983). Unlike in amniotes, the hypothalamic recess in amphibians is very large, and the PVO extends towards the lateral edge of the hypothalamic recess called the lateral recess of the infundibulum (Neary & Northcutt, 1983). We found *th2* ^+^ cells in both the rostral (Figures 6a,b and 7e) and lateral (Figure 7i) edges of the LR. The frontal sections of the rostral PVO are similar to the avian PVO, with abundant *th1* ^+^/TH^+^ non‐CSF‐c cells located dorsolaterally (Figures 1f and 6b,c; asterisks). Unlike the chicken PVO, some TH^+^ CSF‐c cells were observed in the *Xenopus* PVO (Figure 6d–f; arrowheads). We also observe a colocalization of *th2* labeling with TH^+^ immunoreactivity in non‐CSF‐c cells (Figure 6d–f; arrows).

### 3.3 Some CSF‐c cells coexpress *TH2* and *TPH1* and are DA and 5‐HT immunopositive

**Figure 8:**
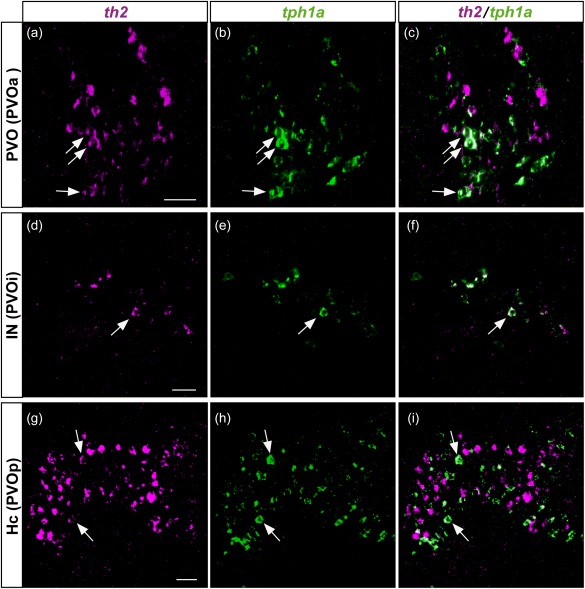
Colocalization of *th2* and *tph1a* in the three CSF‐c cell populations of zebrafish. Frontal sections of the adult zebrafish brain showing the three CSF‐c cell populations, PVO (a–c), IN (d–f), and Hc (g–i), which are also known as PVOa, PVOi, and PVOp, respectively. Single confocal planes show that *th2* (magenta) and *tph1a* (green) are found in the same cell population, and some cells coexpress both of them (arrows). Scale bar = 20 µm

It has been shown that the CSF‐c cells in the hypothalamic region display 5‐HT as well as DA immunoreactivity in many species (Corio, Thibault, & Peute, 1992; Kaslin & Panula, 2001; Meneghelli et al., 2009; Parent & Northcutt, 1982). Although the DA and 5‐HT cell clusters have been considered to be separate cell populations, they are topologically very close, prompting us to investigate whether they could exhibit some colocalization. Double fluorescent in situ hybridization of *TH2* and *TPH1* reveals that indeed some *TH2*‐expressing CSF‐c cells also express *TPH1* in chicken (Figure 7a–d), *Xenopus* (Figure 7e–l), and zebrafish (Figure 8).

In chicken, the *TH2*/*TPH1* colocalization in CSF‐c cells is observed both at a late embryonic stage (E15, data not shown) and at a juvenile stage (4 weeks; Figure 7a–d). Notably, *TH2* and *TPH1* labeling are found in the soma and also in the processes that protrude into the ventricle (Figure 7d).

In *Xenopus*, we detect cells that coexpress *th2* and *tph1* both in the rostromedial (Figure 7e–h) and the caudolateral (Figure 7i–l) parts of the lateral recess. In contrast to the chicken CSF‐c cells, *th2* and *tph1* colabeling is restricted to the cell soma in *Xenopus* (Figure 7h).

In previous works (Yamamoto et al., 2010, 2011), we showed that the zebrafish *th2* is expressed in CSF‐c cell populations in the hypothalamic ventricular areas. We here show that *tph1a*, one of the two *tph1* paralogs duplicated in the teleost lineage (Bellipanni et al., 2002; Gaspar & Lillesaar, 2012), is also expressed in these CSF‐c cell populations (Figure 8), some of them colocalizing with *th2* transcripts (Figure 8; arrows).

Note that the three *th2* ^+^/*tph*1^+^ CSF‐c cell populations in zebrafish are named differently according to the authors (see Discussion). Following the terminology of Rink and Wullimann (2001), we previously called the *th2* ^+^ CSF‐c cell populations the PVO, the intermediate nucleus of hypothalamus (IN), and the caudal zone of periventricular hypothalamus (Hc) (Yamamoto et al., 2010, 2011). In parallel, Kaslin and Panula (2001) named the three 5‐HT cell populations the anterior PVO (PVOa), the intermediate PVO (PVOi), and the posterior PVO (PVOp) (Kaslin & Panula, 2001).

**Figure 9:**
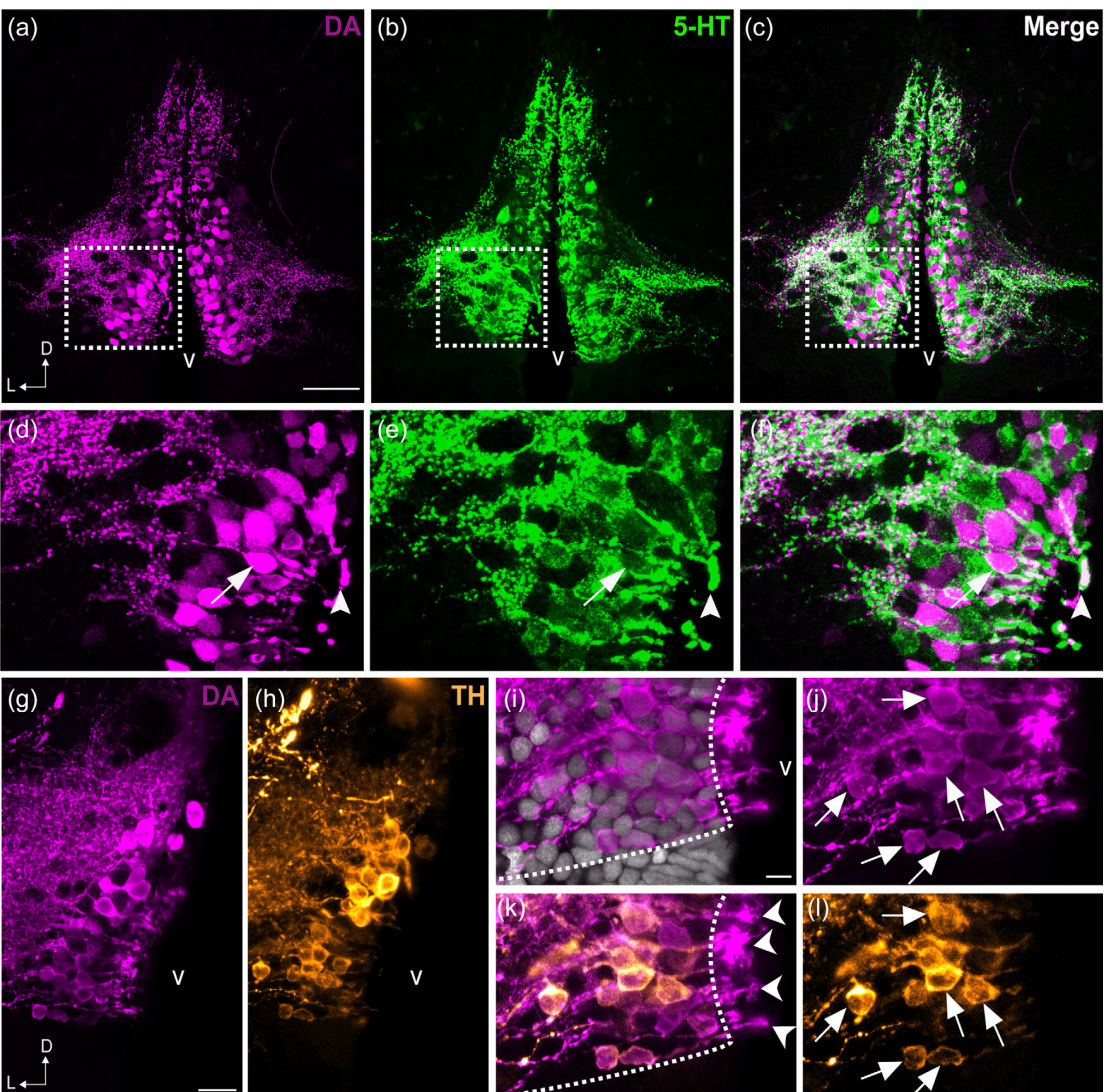
Colocalization of DA and 5‐HT in the PVO cells of zebrafish. Frontal sections of the anterior PVO (PVOa) in adult zebrafish demonstrate that CSF‐c cells are immunoreactive to DA (magenta; a, d) and to 5‐HT (green; b, e). The areas in dashed rectangles in (a), (b), and (c) are shown at higher magnification (Z‐projection = 5 µm) in (d), (e), and (f) respectively. Both monoamines were observed in a few cell bodies (arrow in f) and endfeet (arrowhead in f). In teleosts, some of the DA^+^ CSF‐c cells (g) are also immunoreactive for TH (h; orange). Higher magnification images of the PVO are shown in (i–l**)**. In CSF‐c cells, intense DA immunoreactivity is present in the cell soma (j; arrows), processes, and the endfeet contacting the ventricle (k; arrowheads). In contrast, intense TH immunoreactivity is mostly observed in the soma and processes (l; arrows), but not in the endfeet. D = dorsal; L = lateral; v = ventricle. Scale bar = 50 µm in (a) (applies to a, b, c); 200 µm in (g**)** (applies to g, h); 100 µm in (i) (applies to i, j, k, l)

In agreement with the coexpression of *th2* and *tph1a* genes, we observed that some CSF‐c cells contain both DA and 5‐HT in zebrafish (Figure 9a–f). As previously shown (Yamamoto et al., 2011), many DA^+^/TH^+^ CSF‐c cells are found (Figure 9j,l; arrows), although DA^+^ CSF‐c cells are more abundant than TH^+^ CSF‐c cells. Higher magnification images show that both DA^+^ and TH^+^ cell processes extend into the ventricle (Figure 9i–l), and we observe intense DA immunoreactivity in the terminals protruding into the ventricle (Figure 9k; arrowheads).

### 3.4 CSF‐c cells along the hypothalamic recess are immunonegative for a pan‐neuronal marker, HuC/D

**Figure 10:**
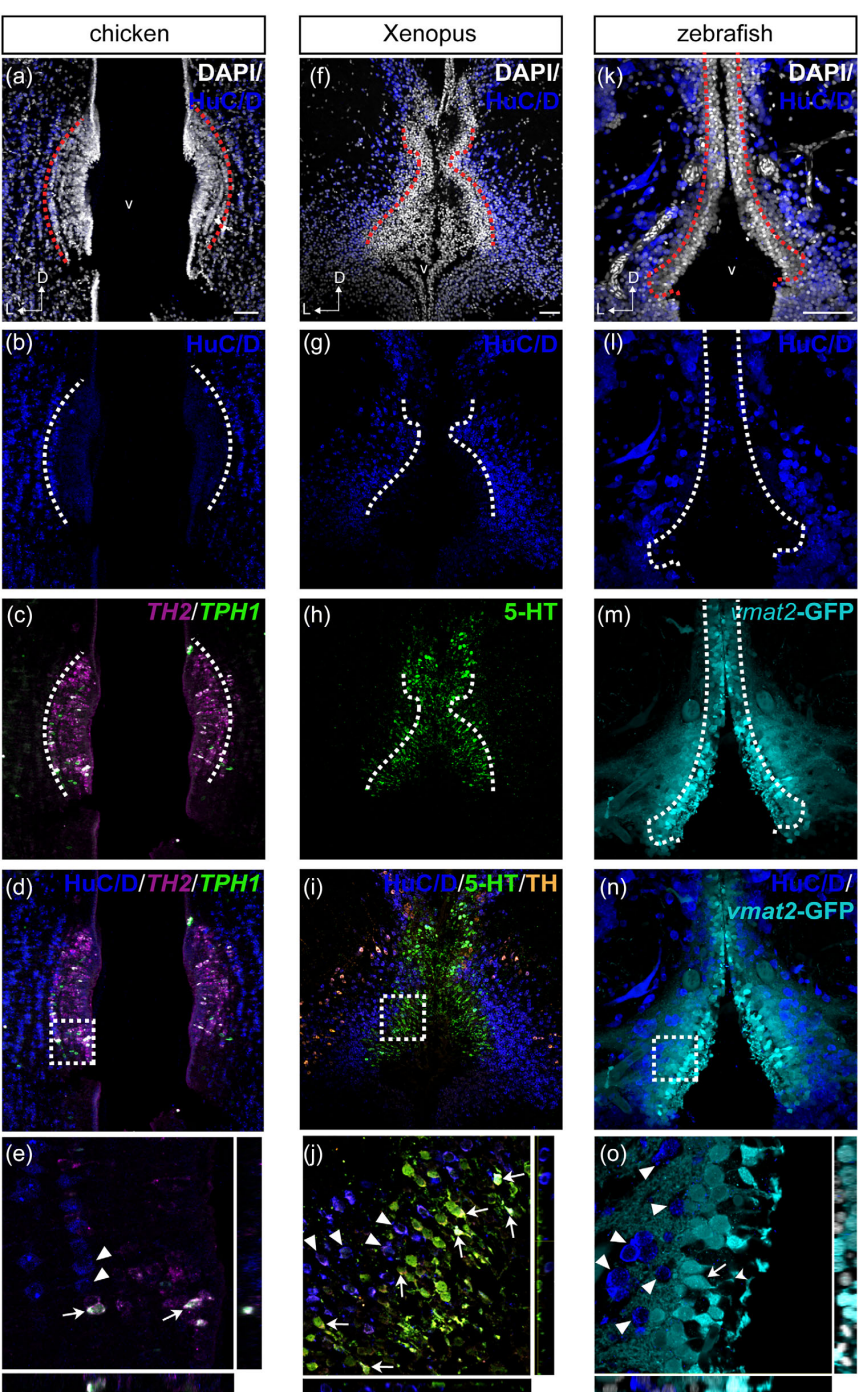
Comparison of HuC/D immunoreactivity in the anterior PVO cell populations in chicken, *Xenopus*, and zebrafish. Frontal sections of the anterior PVO of chicken (a–e), *Xenopus* (f–j), and zebrafish (k–o) are shown. The HuC/D immunoreactivity (dark blue) is absent in the PVO, where monoaminergic CSF‐c cells are located (dashed lines), in chicken (a–d), *Xenopus* (f–i), and zebrafish (k–n). The areas within dashed squares in (d), (i), and (n) are shown at higher magnification in (e), (j), and (o), respectively. *TH2* ^+^/*TPH1* ^+^ CSF‐c cells (white in e; arrows) are not HuC/D^+^ (e; arrowheads) in the chicken PVO. In the *Xenopus* PVO as well, monoaminergic 5‐HT^+^/TH^+^ CSF‐c cells (j; arrows) are not immunolabeled by HuC/D, whereas TH^+^ adjacent to the PVO are HuC/D^+^ (j; arrowheads). Similarly, CSF‐c cells that express GFP in the enhancer trap transgenic line *ETvmat2*:*GFP* (*vmat2*‐GFP; cyan) are HuC/D^‐^ (n). HuC/D^+^ cells (o; arrowheads) are adjacent to *vmat2*‐GFP cells (o; arrow) whose process bathes the ventricle (o; curved arrowhead). D = dorsal; L = lateral; v = ventricle. Scale bar = 50 µm in (a) (applies to a, b, c, d); 50 µm in (f) (applies to f, g, h, i); 50 µm in (k) (applies to k, l, m, n)

**Figure 11:**
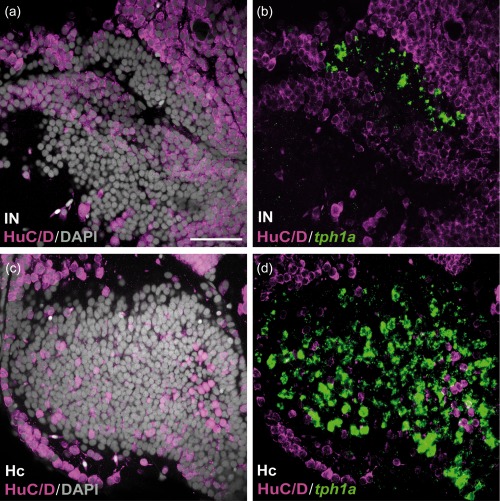
HuC/D immunoreactivity in the caudal CSF‐c cell populations in zebrafish. Frontal sections of the zebrafish IN (a and b) and Hc (c and d), with the midline to the left. Gray represents DAPI staining. The *tph1a* ^+^ CSF‐c cells (green) are negative for HuC/D (magenta). Scale bar = 50 µm (applies to a, b, c, d)

Despite the monoaminergic phenotype, we found that CSF‐c cells are not labeled by HuC/D, which is often used as a marker for differentiated neurons (Okano & Darnell, 1997; Wakamatsu & Weston, 1997). None of the CSF‐c cell markers, *TH2*/*TPH1* transcripts, 5‐HT immunoreactivity, or *vmat2*‐GFP, was colocalized with HuC/D immunoreactivity (Figure 10a–e for chicken, Figure 10f–j for *Xenopus*, Figures 10k–o and 11 for zebrafish).11

The lack of HuC/D immunoreactivity is restricted to the CSF‐c cells, and this is not a feature of all monoaminergic cells, since non‐CSF‐c TH^+^ cells are HuC/D^+^ (Figure 10i,j; arrowheads).

## 4 Discussion

### 4.1 Nomenclature and homology of the CSF‐c cells in jawed vertebrates

CSF‐c cells in the hypothalamic region have been observed in various nonmammalian species except in placental mammals. The abundance and distribution of the CSF‐c cells largely vary depending on animal groups, and there is some confusion in the homology and nomenclature of the CSF‐c cells. In tetrapods, monoaminergic CSF‐c cells around the hypothalamic recess are considered to be a single cell population called the PVO. In *Actinopterygii* (ray‐finned fish which includes the teleost), the CSF‐c cells are much more abundant than in *Sarcopterygii* (lobe‐finned fish which includes the tetrapod), and the nuclei containing the CSF‐c cells are named differently according to the authors or species (sunfish (Parent, Dube, Braford, & Northcutt, 1978), garfish (Parent & Northcutt, 1982), goldfish (Hornby, Piekut, & Demski, 1987; Yoshida et al., 1983), sterlet (Kotrschal, Krautgartner, & Adam, 1985), mormyrid (Meek, Joosten, & Hafmans, 1993)).

In our previous publications describing the *th2* ^+^ DA cell populations in zebrafish (Yamamoto et al., 2010, 2011), we used the nomenclature proposed by Rink and Wullimann (2001), in which the term “PVO” was only applied to the most anterior population, and the two posterior ones were considered to be CSF‐c cell populations located in the intermediate nucleus (IN) and the caudal zone of periventricular hypothalamus (Hc). In contrast, serotoninergic cell populations in zebrafish have been named anterior PVO (PVOa), intermediate PVO (PVOi), and posterior PVO (PVOp), respectively (Kaslin & Panula, 2001).

One‐to‐one homology of CSF‐c cell populations between *Actinopterygii* and *Sarcopterygii* is not yet clear. By focusing on the ventricular organization, the most posterior population (Hc or PVOp) is not likely to be homologous to the tetrapod PVO, since it develops around the posterior recess (PR) which does not exist in tetrapods. The lateral recess (LR) of the *Actinopterygii* corresponds to a lateral expansion of the third ventricle of tetrapods (called the “lateral recess of the infundibulum” in amphibians; Neary & Northcutt, 1983), while the PR is only present in some groups of *Actinopterygii* including teleosts and nonteleost fishes such as gar (Parent & Northcutt, 1982) and sturgeon (Kotrschal et al., 1985). Since PR is neither found in Polypterus (one of the most basal group of *Actinopterygii*; López & González, 2014) nor in *Chondrichthyes* (sister group of *Osteichthyes*; Stuesse, Cruce, & Northcutt, 1994), it is likely that the ancestor of jawed vertebrates did not possess PR.

**Figure 12:**
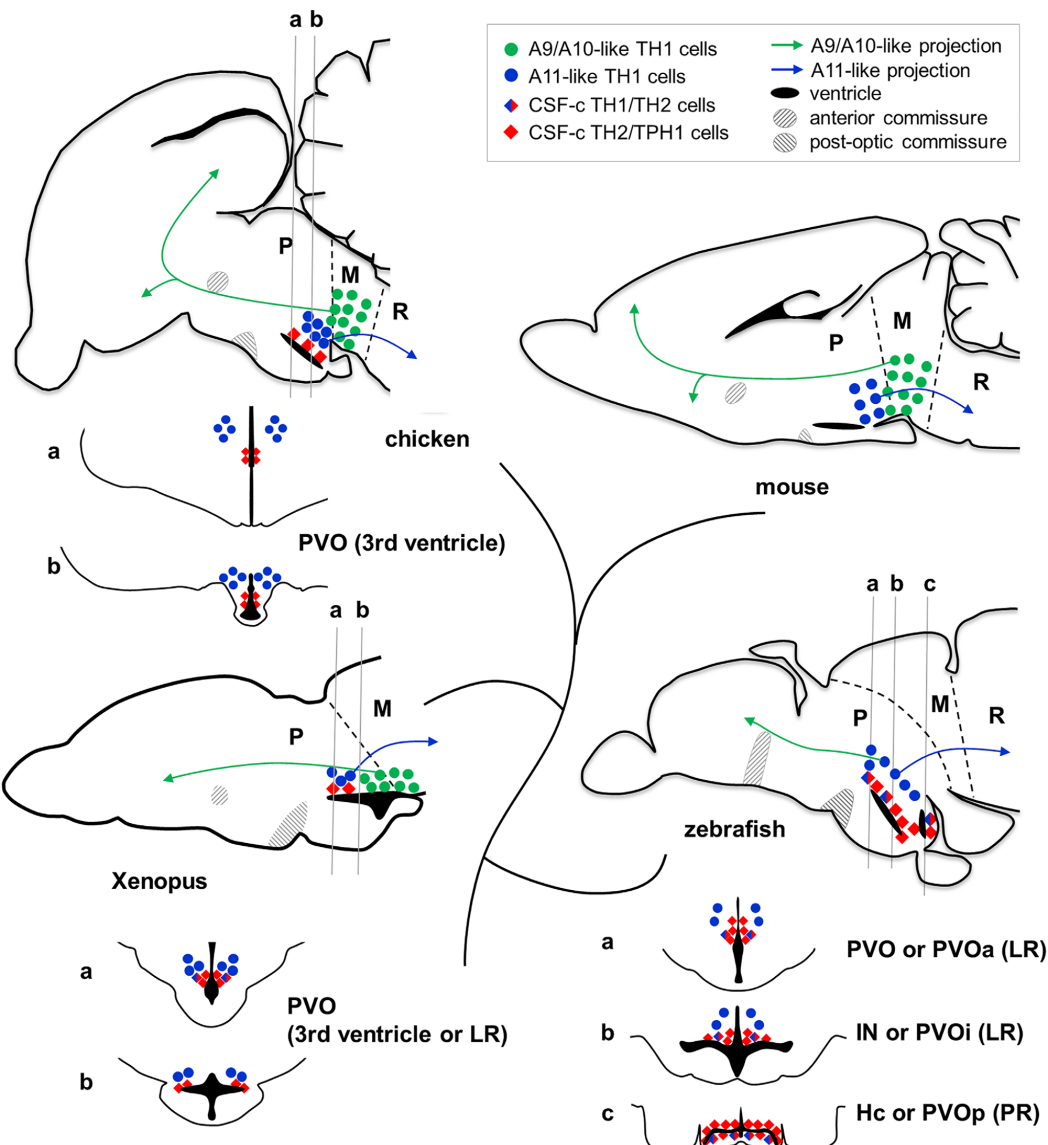
Comparison of monoaminergic cell populations in different groups of *Osteichthyes*. Some comparable monoaminergic cell groups are plotted on schematic drawings of brain sections of mouse, chicken, *Xenopus*, and zebrafish. The sagittal plane is close to the midline (rostral to the left). The diamonds represent CSF‐c cells, and the circles represent non‐CSF‐c cells. *TH2*/*TPH1*‐expressing CSF‐c cells (red diamonds) are commonly found along the hypothalamic recess throughout vertebrates, while placental mammals have lost the CSF‐c cells in the hypothalamic region. Below the sagittal section of chicken, *Xenopus*, and zebrafish, frontal sections around the hypothalamic recess (corresponding to the level of gray lines; a,b,c) are shown. In chicken and *Xenopus*, there is only one hypothalamic recess (named either 3rd ventricle or lateral recess; LR), while in zebrafish, there is an additional recess (posterior recess; PR). Note that in *Xenopus*, the caudally located CSF‐c cells are not observable in the sagittal section close to the midline, because it is located at the lateral end of the LR. The A11‐like *TH1*‐expressing cell population (blue dots; non‐CSF‐c cells) projecting to the spinal cord is commonly found dorsolateral to the CSF‐c cells in all the vertebrate groups. More caudally, the A9/10‐like DA cell population projecting to the telencephalon (green dots) is found in tetrapods, while this cell population is lacking in teleosts. L = lateral recess; M = mesencephalon; P = prosencephalon; PR = posterior recess; R = rhombencephalon

It is ambiguous whether the *Sarcopterygii* PVO is homologous only to the anterior population (“PVO” by Rink and Wullimann, “PVOa” by Kaslin & Panula), or also to the intermediate population (“IN” by Rink and Wullimann, “PVOi” by Kaslin & Panula). Based on the observation of *Xenopus* and zebrafish frontal sections, the rostromedial PVO in *Xenopus* (Figures 7e–h and 10f–j) is similar to the PVO (PVOa) in zebrafish (Figures 8a–e and 10k–o), and the caudolateral PVO in *Xenopus* (Figure 7i–l) is similar to the IN (PVOi) (Figure 8f–j) in zebrafish (also see Figure 12, *Xenopus* and zebrafish). Whether the amphibian PVO consists of a single cell population or two separate populations is a key issue to determine the one‐to‐one homology of the CSF‐c cells. Further investigation, including 3D analysis of the amphibian PVO, will be necessary to elucidate the homology issue.

In any case, the abundance of monoaminergic CSF‐c cells is reduced in the tetrapod lineage as compared to *Actinopterygii*, with the extreme case of complete loss in mammals. The expression of *TH1* in the CSF‐c cells is also reduced in tetrapods, with no *TH1* expression in the avian CSF‐c cells. Since TH^+^ (which is presumably *TH1* ^+^) CSF‐c cells are abundant in many teleosts and *Chondrichthyes* (Smeets & Reiner, 1994), it is likely that the ancestor of jawed vertebrates possessed abundant CSF‐c cells which express both *TH1* and *TH2*. Although the functional significance is not clear yet, it is likely that they played an important role in the brain of ancestral vertebrates.

### 4.2 DA/5‐HT dual phenotype of the hypothalamic CSF‐c cells

It has been long known there are DA^+^ cells in the CSF‐c cells around the hypothalamic area, although these cells are not TH immunoreactive. Thus, these DA^+^ cells were considered to be DA‐accumulating cells (Smeets & Reiner, 1994). As a matter of fact, the presence of the *TH2* transcripts in these cells strongly suggests that DA is synthesized by the TH2 enzyme in this cell population.

Moreover, we found the CSF‐c cells containing both DA and 5‐HT. The localization of *th2* in 5‐HT^+^ cells devoid of TH immunoreactivity prompted some authors to propose that TH2 is functionally equivalent to TPH, synthesizing 5‐HT instead of DA (Ren, Li, Zhong, & Lin, 2013). In their hypothesis, TH1 expressing cells synthesize DA while TH2 expressing cells synthesize 5‐HT in zebrafish. However, later studies do not support this idea (McPherson et al., 2016; Semenova, Chen, Zhao, Rauvala, & Panula, 2014). Based on our results revealing the presence of *TH2* (but not necessarily *TH1*) in the DA^+^ CFS‐c cells in three vertebrate groups, it is more likely that TH2 is able to synthesize DA in this area.

A difference between the data of Semenova et al. (2014) and this study is that the former did not find colocalization of TH2 and 5‐HT, thus claiming that there is no colocalization of DA and 5‐HT. The discrepancy may come from the use of newly developed anti‐TH2 antibody instead of an anti‐DA antibody as we did. In addition, it is possible that the percentage of the cells cocontaining DA and 5‐HT could be variable depending on physiological conditions. This is the case for the colocalization in turkey of DA and melatonin (MEL), another indolamine synthesized from 5‐HT with the additional enzyme arylalkylamine *N*‐acetyltransferase (AANAT). The respective levels of DA and MEL fluctuate depending on the circadian rhythms that influences seasonal reproduction (Kang et al., 2010, 2007).

Similarly to the case of the *TH2* gene, which has been long ignored due to its loss in the mammalian genome, the importance of *TPH1* in the brain has been also underestimated. This is because *TPH1* expression is found only in the pineal gland that synthesizes MEL in mammals. Studies on nonmammalian species suggest that the presence of dual neurotransmitter phenotypes of catecholamines and indolamines in the forebrain is common in *Osteichthyes* except for mammals.

### 4.3 Physiological roles of the monoaminergic CSF‐c cells

A study in quail revealed that 5‐HT cells in the avian PVO express opsin 5, a UV sensitive opsin, suggesting that the PVO is involved in deep brain photoreception that regulates seasonal reproduction (Nakane et al., 2010). Since many *TPH1* ^+^ cells coexpress *TH2* in the chicken brain, it is possible that the dual DA/5‐HT phenotype plays a significant role in this function. Indeed, it has long been proposed that hypothalamic CSF‐c cells play a role in deep brain photoreception to synchronize periodicity in nonmammals (Vigh et al., 2002). The loss of CSF‐c cells may thus be correlated to the loss of deep brain photoreception in the mammalian forebrain.

A recent publication in zebrafish larvae suggests that the *TH2* ^+^ DA cells in Hc modulate the initiation of swimming behavior (McPherson et al., 2016). As Hc would have evolved only in the *Actinopterygii*, it is possible that the hypothalamus in teleosts exerts functions that do not exist in tetrapods.

### 4.4 Homology of the DA cell populations in the hypothalamus and surrounding regions

Based on the current and previous studies, different categories of monoamine cells can be represented as shown in Figure 12. In birds, amphibians, and teleosts, *TH2*/*TPH1* coexpressing cells, that is, DA/5‐HT cocontaining CSF‐c cells (Figure 12; red diamonds) are present around the hypothalamic recesses (third ventricle or LR). The posterior recess (PR) and the surrounding DA^+^/5HT^+^ CSF‐c cells (Hc or PVOp) are observed only in some groups of *Actinopterygii* including teleosts (Figure 12; zebrafish).

Since there is no DA^+^ CSF‐c cells in the mammalian forebrain, those found in nonmammalian species would not be homologous to any of the mammalian DA cell populations. The hypothalamus has evolved in a divergent manner in mammals and teleosts. Mammals have lost the DA^+^ CSF‐c cells, while teleosts underwent a significant increase in the relative number of these cells as compared to other *Osteichthyes*. Thus, these two animal groups must be compared with caution.

Based on a one‐to‐one comparison of zebrafish and mammalian DA cell populations, it has been proposed that DA cells in the PVO (PVOa) are homologous to the A12/A14 DA population (Filippi, Mueller, & Driever, 2014). This is unlikely, since mammalian A12/A14 do not comprise CSF‐c cells. Furthermore hypothalamic A12 cells in amniotes project to the median eminence and regulate pituitary functions via portal blood system, while the teleost pituitary receives direct DA inputs from the anterior optic recess region (ORR) (Fontaine et al., 2015). Thus, it is possible that teleosts do not have DA cells homologous to the mammalian A12 group.

Dorsolateral to the CSF‐c *TH2* ^+^/*TPH1* ^+^ cells, non‐CSF‐c *TH1* ^+^ cells are consistently found throughout *Osteichthyes* (Figure 12; blue circles). They are characterized by a distal projection to the spinal cord (Figure 12; blue arrows; Medina, Puelles, & Smeets, 1994; Sánchez‐Camacho, Marín, Smeets, Donkelaar, González, 2001; Tay, Ronneberger, Ryu, Nitschke, & Driever, 2011) as the mammalian A11 cell group does (Björklund & Skagerberg, 1979; Skagerberg, Björklund, Lindvall, & Schmidt, 1982). Nonetheless the homology of this cell population needs to be determined carefully, because there are some discrepancies. First, in teleosts, which lack mesencephalic DA cells, these non‐CSF‐c TH1 cells were also suggested to be similar to A9/A10, based on their projection to the subpallium (Figure 12; green arrow in zebrafish; Rink & Wullimann, 2001). Second, the regional identity of the corresponding area is not clear. In amniotes it is often considered to be the hypothalamus (which is a part of the secondary prosencephalon), while in teleosts it is considered to be the posterior tuberculum (which is a part of the diencephalon). A recent publication in zebrafish (Biran, Tahor, Wircer, & Levkowitz, 2015) proposes to reconsider this part of the posterior tuberculum as a part of the hypothalamus, as defined in mammals (Puelles & Rubenstein, 1993, 2003). A boundary between the hypothalamus and the diencephalon remains to be more precisely defined.

The current model of the hypothalamic organization is largely based on mammalian studies. However, our study demonstrates that the mammalian hypothalamus is an exceptional case lacking a whole population of CSF‐c cells. To better understand the general organization in vertebrates, models of hypothalamic organization need to be refined by including a larger range of vertebrate species.

## Supporting information

Supporting Information Video 1.

Supporting Information Video 2.
